# Natural resources, financial development and structural transformation in Sub-Saharan Africa

**DOI:** 10.1016/j.heliyon.2023.e19522

**Published:** 2023-08-30

**Authors:** Chinazaekpere Nwani, Benedette Nneka Okezie, Anthony Chukwuma Nwali, Johnson Nwokeiwu, Gloria Ifeoma Duruzor, Ogbonna Nweze Eze

**Affiliations:** a Department of Economics and Development Studies, Alex Ekwueme Federal University, Ndufu-Alike, Ebonyi State, Nigeria; b Accountancy Department, Alex Ekwueme Federal University, Ndufu-Alike, Ebonyi State, Nigeria; c Department of Business Administration, Alex Ekwueme Federal University, Ndufu-Alike, Ebonyi State, Nigeria

**Keywords:** Natural resources, Structural transformation, Industry structure, Financial development, Sub-Saharan Africa

## Abstract

Structural transformation is a crucial prerequisite for inclusive and sustainable development. In this context, this study examines the influence of natural resource dependence on the transformation of industrial structure in a panel of 30 Sub-Saharan (SSA) economies from 1995 to 2019, taking into account the role of financial development. The empirical analysis relies on the newly developed bias-corrected method-of-moments estimator. The findings indicate that natural resource dependence leads to a reduction in the output contribution of the manufacturing and service industries. On the other hand, financial development through the institutions channel decreases the output value of extractive industries while increasing the output value of manufacturing and service industries, resulting in a net positive effect on the evolution of industrial structure away from extractive-based industrialization. Considering the complex multidimensional nature of financial development, further findings suggest that the depth and efficiency of financial systems are critical areas for deepening structural diversification in the sub-region. Additionally, the findings reveal a U-shaped relationship between per capita GDP and industrial structure transformation. These findings highlight that natural resource dependence inhibits structural transformation in SSA and emphasize the importance of enhancing the depth and efficiency of financial systems as part of strategies to break the resource curse.

## Highlights

• Industrial structure in Sub-Saharan Africa countries
• The role of economic dependence on natural resources
• Transformation-inducing effect of financial development

## 1 Introduction

Sub-Saharan Africa (SSA) is rich in natural resources, boasting significant reserves of minerals and hydrocarbons. Countries like Nigeria, Angola, and Gabon are among Africa's largest oil producers, while central and southern Africa possess abundant reserves of gold and diamonds. The average share of total natural resources rents in gross domestic product (GDP) during the period 1990–2020 stood at 10.03% [1]. Given these abundant natural resources, there was hope that the continent would experience a growth “take-off” [2]. However, this perspective has been challenged by various studies revealing the existence of the resource curse in most SSA countries [3,4]. The resource curse manifests as slower economic growth, higher poverty rates, increased social inequality, and heightened political instability [3,4]. Another manifestation is the Dutch Disease, which negatively affects the macroeconomy by reducing competitiveness in non-resource sectors and neglecting other industries [2]. This present study re-examines the symptoms of the resource curse in SSA from the perspective of industrial structure transformation, while also capturing the role of financial development.

The driving force behind structural transformation is the productivity of the modern sector, which includes manufacturing and services [5]. According to estimates from the World Bank [1], the share of manufacturing in GDP, which averaged 16.13% in 1990, declined to 13.93% in 2000 and further decreased to 11.17% in 2020. Services in GDP, which reached 53.5% in 2015, experienced a steady decline afterwards, settling at 49.4% in 2020. On the other hand, the mining, construction, and utilities sectors dominate the output of the secondary industry, with their share in GDP increasing from 13.57% in 1990 to 14.78% in 2000 and further to 15.29% in 2020. Additionally, there has been a growing contribution from the primary industry, with the share of agriculture, forestry, and fishing in GDP increasing from 15.48% in 2000 to 18.48% in 2020. These figures reveal a decline in the manufacturing sector's contribution to GDP and a growing output from the agriculture, mining, construction, and water and energy utilities sectors. This indicates the presence of productivity gaps in the region. Existing literature suggests that shifting from a subsistence/agrarian and resource-extractive economy to a more manufacturing and services-oriented one plays a crucial role in driving economic growth and enhancing productive capacity [5,6]. Manufacturing, in particular, offers unique advantages such as capital accumulation, economies of scale, and technological advancements, making it a preferred avenue for improving productivity [6]. Furthermore, manufacturing has significant spillover effects on the services sector through productive linkages, generating externalities in technology development, skill acquisition, and learning, which are essential for enhancing overall competitiveness [6].

The skewed composition of industrial output towards non-manufacturing sectors offers another perspective to understand the symptoms of the resource curse in the SSA economies. One manifestation of the Dutch disease phenomenon is the crowding out effect, which suggests that reliance on natural resources can hinder the development of high-tech manufacturing and tertiary industries [[7], [8], [9]]. This occurs because the influx of low-tech mineral extractive industries can dominate the economic landscape, diverting resources and investment away from other sectors [10]. When an economy becomes heavily dependent on mineral extractive industries, substantial resources, infrastructure, and labour tend to be allocated to these sectors. Consequently, there may be limited incentives and investment for the growth of high-tech manufacturing and tertiary industries [6]. Moreover, mineral extractive industries may not significantly contribute to the overall productive capacity of an economy beyond their immediate output [11]. Typically, these industries involve the extraction and exportation of raw materials without substantial value addition within the region [11]. This limitation can hinder diversification efforts and the development of industries with higher value-added activities.

The essence of industrial structure transformation lies in the movement of production factors from low-productivity sectors to resource-efficient and high-productivity sectors, with the optimal allocation and coordination of these factors across different sectors being the underlying mechanism [12]. The interaction between the financial sector and the real sector is widely recognized as a crucial policy tool [13]. Accordingly, SSA countries have intensified policy efforts over the past four decades to develop their financial systems. These policy efforts primarily aim to enhance the intermediary functions of the financial system in the region through liberalization, privatization, and the implementation of auxiliary financial markets and sector-specific banking policies [14]. The benefits of these initiatives continue to be debated among experts, particularly regarding the system's capacity to operate efficiently. Structurally, there are three mechanisms through which financial development plays a role in catalyzing economic and structural diversification: diverting financial resources away from less productive sectors, funding activities in more productive sectors, and expanding financial access [14]. These mechanisms correspond to the key dimensions of financial development, namely depth, efficiency, and access. Svirydzenka [15] demonstrates that SSA economies have improved the size (depth) of their financial systems over the past four decades but still exhibit low scores in major efficiency indicators. As policymakers in these economies continue to intensify efforts to enhance the interaction between the financial sector and the real economy, they recognize that improving the allocation efficiency of the system is crucial for catalyzing broader economic efficiency and structural transformation [14].

The empirical literature exploring the impact of natural resource dependence on industrial structure dynamics in SSA is continuously evolving, uncovering the presence of knowledge gaps. One such gap pertains to reliance on a three-sector classification in existing studies, combining the output contribution of all secondary industrial production. For example, studies like Asiamah et al. [2] have adopted this approach, while others have primarily focused on analyzing the output value of the manufacturing sector, as seen in Itaman and Awopegba [16]. However, these previous empirical models have overlooked the independent effects of non-manufacturing industrial sectors such as mining, construction, and utilities when tracing the progress of industrial structure transformation in SSA. To gain deeper insights into the resource curse symptoms, extending previous studies to incorporate the significant contribution of mining, construction, and utilities in SSA is crucial. Another knowledge gap in the literature pertains to the limited empirical research on the structural-transformational effects of financial development, with less emphasis on SSA specifically. To the best of our knowledge, only Itaman and Awopegba [16] have examined the specific case of an SSA economy, particularly Nigeria. Additionally, the existing empirical studies in this area have predominantly focused on the depth of credit supply from the banking system as the sole indicator of financial development. Consequently, these studies have neglected the policy relevance of the multidimensional nature of financial development, which encompasses factors such as depth, access, and efficiency within the institutions and market segments of the financial system. By considering these dimensions, valuable insights and diverse policy directions related to financial development can be derived.

In order to address the aforementioned knowledge gaps in the literature, we have formulated a model that examines the relationship between industrial structure dynamics and economic dependence on natural resources, taking into account the multidimensional characteristics of financial development. To enhance the empirical value of our study, we integrate the traditional non-linear curve that explains the connection between per capita GDP and industrial structure dynamics [5]. This empirical step allows us to explain the shape of the curve, whether it follows a U-shaped or inverted U-shaped pattern, as SSA progresses along the development ladder. This study adds at least two more contributions to the existing literature. First, we disaggregate the output of the secondary industry to analyze the independent impact of non-manufacturing activities, such as mining, construction, and utilities supply. Based on these disaggregated estimates, we construct a metric that tracks the degree of economic activity reallocation from primary, mining, and construction sectors to the manufacturing and tertiary sectors, which have a greater potential for growth. By taking this empirical step, we provide another perspective for understanding the symptoms of the resource curse in SSA. Second, we delve into the three dimensions of financial development, namely depth, access, and efficiency, to understand their role in explaining industrial structure transformation in SSA. Through this empirical step, we aim to determine whether financial development can effectively adjust the impact of natural resource dependence on industrial structure transformation by facilitating capital mobilization, enhancing resource allocation, and expanding financial services. This study will have policy implications for fostering broader economic transformation necessary for promoting growth in resource-efficient and high-productivity sectors in SSA.

The remainder of this paper is structured as follows: Section 2 provides a review of the literature, focusing on the theoretical and empirical background. In Section 3, the data and modeling techniques are described. Section 4 presents the results and their discussion. Finally, Section 5 concludes the paper with some policy remarks.

## 2 Theoretical considerations and literature review

This section covers two main aspects of the literature. Firstly, it examines the theoretical foundations that form the basis for developing hypotheses regarding the impact of natural resources and financial development on industrial structure transformation. Secondly, it evaluates previous empirical studies to highlight research gaps in this field.

### 2.1 Theoretical background and research hypothesis

Structural transformation refers to the long-term composition and reallocation of economic activity across different sectors of the economy, primarily agriculture, industry, and services [[17], [18], [19]]. Early studies, based on stylized facts from advanced countries, demonstrate a sequential shift from agriculture (primary sector) to industry (secondary sector), followed by a subsequent shift from industry to services (tertiary sector) [[20], [21], [22]]. However, these patterns of sectoral composition and aggregate output shifts can be influenced by various factors [17,18,23,24]. Among the commonly considered factors is the degree of economic dependence on natural resources [2,9,25,26]. The income generated from resource extraction can fulfill the import demand for other goods and services, making it challenging to diversify into manufacturing of tradable goods outside the resource sectors [27]. Consequently, the composition and transformation of industrial structure in many developing countries have been analyzed under the framework of the resource curse hypothesis [2].

One of the key symptoms of the resource curse is the Dutch disease, characterized by windfall income and sustained real appreciation of the domestic currency exchange rate. This leads to the expansion of the natural resource segment within the tradable sector, accompanied by a contraction of the non-resource tradable sector, particularly manufacturing [9,[28], [29], [30], [31], [32]]. These symptoms contribute to the dominance of the natural resource sector, which displaces productive resources from other sectors, thereby limiting the scope for manufacturing industries and other productive sectors and leading to an excessive reliance on extractive industries [31,33,34]. Building on this context, Pasaribu [24] argues that the Dutch disease impedes the reallocation of economic activity from extractive sectors to more growth-enhancing manufacturing and tertiary production, thereby prematurely halting the process of structural transformation. Considering the growing significance of export-oriented manufacturing and the emergence of digital technologies that enhance the tradability of services, Alssadek and Benhin [35] and Hasanov et al. [36] contend that economic dependence on natural resources, through the symptoms of the Dutch disease, inhibits the scale, innovation, and spillover effects necessary for triggering structural transformation, as observed in industrialized countries. Consequently, the following hypothesis is formulated to guide this study.

H1 Natural resource dependence has a significant negative impact on industrial structure transformation.

Economic diversification is commonly proposed as the primary strategy to counter the resource curse in developing countries [27,29]. The underlying idea is to expand the productive capacity of sectors other than natural resources. Chang et al. [29] suggest strengthening the links and positive productivity effects between the tradable natural resource sector and the rest of the economy, enabling significant resource rents to contribute to the growth of other productive sectors. In line with this perspective, policymakers have relied on the resource re-allocation effect of financial systems [10,37,38]. According to economic theory, a developed financial system offers benefits such as mobilizing and pooling savings, allocating resources to productive sectors through low-cost trading and information facilitation mechanisms, and supporting diversification and risk mitigation [13]. By influencing savings and investment decisions, financial development directs financial flows toward the most productive sectors [36].

To provide a theoretical framework, Saborowski [13] hypothesizes that a developed financial system restricts capital inflows into low-productivity sectors like construction and mining, thus mitigating the structural consequences of the Dutch disease. Choi [39] argues that it alleviates credit constraints, leading to resource reallocation that stimulates activity in more innovative industries. Beck [40] hypothesizes that it leverages economies of scale to create a competitive advantage in manufacturing and the tradable sector, compensating for technological differences. Feng and Wu [41] suggest that financial development increases investment scale, promotes innovation, and intensifies spill-over effects that drive structural transformation. Building on this body of literature, Xu and Tan [38] propose that financial development has the potential to shape the composition of industrial structure in resource-dependent countries by redirecting financial resources from surplus natural-resource-related sectors towards productive investments in the tertiary industry. Itaman and Awopegba [16] argue that financial development can catalyze the expansion, upgrading, and formation of entirely new tertiary sectors in resource-rich nations. Therefore, the following hypothesis is formulated to guide this study.

H2 Financial development has a significant positive impact on industrial structure transformation.

The provision of financial services is facilitated by a network of financial institutions and markets [39]. Furthermore, the nature of the system can be described using three parameters: the depth of the system, which encompasses its size and liquidity resulting from the mobilization and allocation of financial resources; the capacity to expand access to financial resources; and its resource allocation efficiency [39]. Choi [39] demonstrates that, in addition to increasing the system's depth, enhancing its efficiency plays a vital role in scaling productivity. It enables the flow of capital to industries with high growth potential and fosters investment in sectors that drive structural transformation. Therefore, it can be inferred that considering the diverse characteristics that describe the development of financial systems will provide valuable insights for guiding policy considerations.

### 2.2 Related existing empirical literature

Several empirical studies have delved into the determinants of industrial structure composition and the patterns of transformation [9,17,24,[42], [43], [44]]. These studies collectively demonstrate that long-term changes in industrial structure are shaped by a combination of economic, social, and institutional factors. Notably, factors such as growth in per capita income, economic dependence on natural resources, globalization, and technology transfer have received significant attention in explaining these trends. However, the empirical modeling approaches have exhibited some variations. Initially, studies formulated a linear relationship between per capita GDP and industrial dynamics and subsequently expanded the equation to incorporate the influence of other factors. Specifically, there have been empirical endeavors that incorporated the impact of natural resources and financial development on the dynamics of the industrial structure [24,44], which are particularly relevant to this present study.

Haraguchi et al. [44] examined the role of natural resources in explaining the patterns of changes in manufacturing output in developing countries during the period 1970–2014. The results showed that natural resources inhibit the progress of rapid industrialization by crowding out other productive sectors. Pasaribu [24] used a global sample of 149 countries to test the impact of natural resources on industrial value-added over the period 1970–2014. The results demonstrated that increases in the economic contribution of natural resources have a negligible positive impact on the output value of the tradable manufacturing sector, which rejects the Dutch disease hypothesis. Alssadek and Benhin [35] used data from 1970 to 2016 to examine the resource curse symptoms in a selection of oil-rich economies. The findings revealed that increases in oil production and income have a considerable negative influence on the output contributions of the primary, industrial, and tertiary sectors. Amiri et al. [43] modeled the shift from industry to services in a selection of 28 countries with significant reliance on natural resources using data from 2000 to 2016. The results showed that natural resources inhibit structural shifts in the industrial structure composition, but the effect diminishes as the quality of institutions improves. Studies such as Asiamah et al. [2] and Nkemgha et al. [45] found evidence to support the validity of the resource curse in SSA. Using data from 2005 to 2019 and a dynamic panel-data model, Asiamah et al. [2] showed that economic reliance on natural resources has a negative impact on the growth of the manufacturing and services sectors in the region. Using a different sample from 2000 to 2016, Nkemgha et al. [45] confirmed the negative impact of natural resource dependence on the manufacturing value addition to aggregate output in the region.

Jiang et al. [46] used Chinese provincial data from 2008 to 2016 to examine the impact of financial development on industrial structure transformation. The results showed that the deepening of financial intermediation strengthens the shift from industry to services in the eastern and western provinces but has no significant impact in the central China. Using data from 2008 to 2020, Wang and Wang [47] demonstrated that banking sector development has a higher beneficial influence on the output value of the tertiary industry, contributing significantly to the shift from industry to services in China. Xu and Tan [38] showed that by redistributing resources in the real sector, banking sector development improves the efficiency of natural resource use and thus contributes positively to the upgrading of the industrial structure in China. Alagidede et al. [48] approached the analysis from the standpoint of globalization, discovering that from 1985 to 2015, international financial flows increased the output value of the primary and tertiary sectors while generating insignificant activity in the secondary sectors in SSA. Using Taiwanese firm-level data from 1990 to 2016, Choi [39] showed that financial development favourably skews aggregate output towards the more productive sectors by reducing credit constraints of innovation-based enterprises. In another study, Hasanov et al. [36] showed that deepening financial systems' credit allocation to the private sector significantly mitigates the resource curse symptoms in Azerbaijan and Kazakhstan by increasing the output of the non-oil sectors.

There is a distinct group of empirical studies that have adopted a non-linear approach to exploring the relationship between per capita GDP and industrial dynamics. These studies extended their equations to encompass the influence of various factors, including natural resources. Drawing on some stylized facts observed across countries and over time, these studies argue that the transformation of industrial structure follows either a U-shaped or an inverted U-shaped pattern in relation to per capita GDP (see Tregenna and Andreoni [49], for more details on these stylized facts). These non-linear patterns imply that the transition from a subsistence/agrarian economy to one characterized by manufacturing and services varies at different stages of economic growth. For example, Dabla-Norris et al. [11] conducted a study examining a sample of developed and developing countries spanning from 1970 to 2010. Their findings revealed a U-shaped relationship between per capita GDP and agricultural output, an inverted U-shaped relationship between per capita GDP and manufacturing, and a monotonically increasing relationship between per capita GDP and services. Moreover, they found that financial development has a negative impact on agricultural output and a positive influence on manufacturing and services at lower and higher quantiles, respectively.

In another study focusing on manufacturing output in Nigeria from 1981 to 2018, Itaman and Awopegba [16] introduced a non-linear equation that extended to elucidate the impact of trends in the domestic financial system on industrial progress. The results indicated an inverted U-shaped association between the share of manufacturing in aggregate output and per capita GDP. Additionally, they observed a negative (declining) relationship between the development of the financial (banking) sector and manufacturing output in Nigeria. Furthermore, Botta et al. [5] examined a sample of 36 countries, categorized into developed economies and emerging and developing economies (EDEs), from 1980 to 2017. Their study investigated the influence of per capita GDP, foreign capital inflows, and natural resource rents on the dynamics of manufacturing output. The results showed an inverted U-shaped curve between manufacturing output and per capita GDP in EDEs, while a U-shaped curve was observed in developed economies. The analysis of the overall sample and EDEs indicated that natural resources reduce the manufacturing share in aggregate output, and the effect of foreign capital inflows was negative and significant only in Africa and Latin America.

Theoretical and empirical contributions clearly highlight the need to gain deeper empirical insights into the impact of natural resource dependence and financial development on industrial structure transformation. This holds particular significance for the SSA region, where a substantial share of industrial output is derived from the mining, construction, and utilities sectors. Moreover, the recent financial sector reforms in SSA economies present a valuable opportunity to expand our empirical knowledge and provide evidence-based guidance for policy decisions.

## 3 Empirical strategy

### 3.1 Construction of empirical model

Building upon the works of Dabla-Norris et al. [11], Itaman and Awopegba [16], and Botta et al. [5], this study formulates an extended model that explores the influence of natural resources and financial development on the dynamics of industrial structure in the SSA region, incorporating additional explanatory variables:

(1) $${DSD}_{i.\;t}=\alpha_{0}+\alpha_{1}{DSD}_{i,t-1}+\alpha_{2}{NR}_{i,t}+\alpha_{3}{FD}_{i,t}+\alpha_{4}{Pgdp}_{i,t}+\alpha_{5}{Pgdp}_{i,t}^{2}+\alpha_{6}{TrGl}_{i,t}+\alpha_{7}{FinGl}_{i,t}+\varepsilon_{i,t}\;;\;t=1,.\;.\;.,T;\;i=1,.\;.\;.,N;\;\varepsilon_{i,t}=\mu_{i}+u_{i,t}$$

where TandN define the time and number of countries in the sample, respectively. DSD represents the vector of industrial structure indicators, and ${DSD}_{i,t-1}$ is the lag of DSD. NR represents economic dependence on natural resources, while FD is a measure of financial development. The slope coefficient $\alpha_{2}$ explains the effect of NR on DSD. Consistent with the RC hypothesis, a negative and statistically significant $\alpha_{2}$ will indicate that economic dependence on natural resources inhibits structural transformation [9]. Similarly, $\alpha_{3}$ explains the effect of FD on DSD, and a positive and statistically significant estimate will suggest that financial development enhances structural transformation. Other parameters in Eq. (1) include $\alpha_{0}$, the intercept of the model, and $\varepsilon_{i,t}$ the idiosyncratic error term which is further defined by $\mu_{i}$ for the time effects and $u_{i,t}$ as the error term. $\alpha_{1}$ provides the initial conditions or convergence of DSD among the countries. A positive $\alpha_{1}$ would indicate divergence of industrial structure dynamics. Eq. (1) controls for the impact of per capita GDP (Pgdp) and economic globalization (Gl) on DSD indicators. By accounting for their influence, we aim to capture the effects of other factors that shape the patterns of industrial structure transformation [5, 11, 16].

Per capita GDP reflects the level of economic progress [50]. As per capita income increases, the economy evolves, allowing for significant shifts in the sectorial composition of aggregate output [17]. It is possible that changes in Pgdp are altering the consumption and expenditure patterns of economic units in SSA economies, thereby shaping the process of structural transformation in the region. Dabla-Norris et al. [11], Itaman and Awopegba [16] and Botta et al. [5] hypothesized a non-linear relationship between Pgdp and DSD, suggesting that the effect of Pgdp could vary at different stages of growth. Hence, Eq. (1) is specified to include both Pgdp and its quadratic term, Pgdp^2^. This enables us to examine the shape of the curve between Pgdp and DSD. A statistically significant negative $\alpha_{4}$ and positive $\alpha_{5}$ would indicate a U-shaped curve, implying that DSD decreases at lower levels of Pgdp and increases at higher levels. An inverted U-shaped curve is also possible, requiring a positive $\alpha_{4}$ coefficient and a negative $\alpha_{5}$ coefficient [49]. Additionally, the turning point level of Pgdp can be determined by calculating ${-\alpha }_{Pgdp}/2\alpha_{{Pgdp}^{2}}$ [16]. SSA economies have experienced increased integration with the global economy in recent years. Industrial tariffs are at their lowest levels, and foreign direct investment flows have reached new heights. These conditions can facilitate technology transfer and create markets for industrial goods and services [17]. Following Botta et al. [5], we hypothesize diverse consequences of economic globalization depending on the degree of integration into the global economy through trade flows (TrGl) and financial flows (FinGl).

### 3.2 Estimation method

The functional relationship specified in Eq. (1) incorporates the lagged dependent variable (${DSD}_{i,t-1}$). This suggests a potential correlation between the factors driving shifts in industrial structure composition and the error term [51]. Consequently, using the traditional “fixed-effects” (FE) /“random-effects” (RE) estimators for Eq. (1) would lead to biased estimates [51]. To address this issue of dynamic panel bias, several approaches can be employed. One approach is to estimate the model using instrumental variables (IV) and generalized method of moments (GMM) estimators. Among the various techniques available in this category, the System GMM method, proposed by Arellano and Bover [52] and Blundell and Bond [53], is commonly used in empirical applications. As pointed out by Rodman [54], the System GMM estimator is particularly efficient when modelling panels with a small number of time dimensions (T) and a large number of cross-section dimensions (N). For moderately large-T panels, Norkute et al. [55] proposed an IV estimator known as IV-DFreg, which efficiently addresses the distortion effects [56]. As an alternative to System GMM and IV-DFreg techniques, Breitung et al. [57] introduced the bias-corrected method-of-moments (BC-MM) estimator. This estimator is capable of producing unbiased and reliable parameter estimates for both small-T and large-T panels [57].

The panel dataset used in this study has a moderately large sample size, with T = 25 and N = 30. Consequently, the analytical form of the BC-MM estimator is employed in this study. The BC-MM estimator adjusts the moment conditions of the dynamic panel model specifications. One advantage is that it does not require a preliminary consistent estimator. Additionally, it can handle both FE and RE assumptions, as well as heteroskedastic errors, cross-sectional dependence and higher-order autoregressive models [57]. Through Monte Carlo simulation, it has been demonstrated that the BC-MM estimator outperforms other competing techniques and produces more efficient parameter estimates than the System GMM estimator [57]. The decision between fixed-effects (FE) and random-effects (RE) is determined using the Hausman specification test. Table A1 in the Appendix presents the results, showing that FE is a more efficient estimator for the various specifications of Equation (1). Therefore, the estimation of Equation (1) utilizes the fixed-effects version of the BC-MM estimator (FE-BC-MM) as follows:

(2) $${DSD}_{i.\;t}=\alpha_{0}+\sum_{j=1}^{p}\alpha_{j}{DSD}_{i,t-j}+\alpha_{2}{NR}_{i,t}+\alpha_{3}{FD}_{i,t}+\alpha_{4}{Pgdp}_{i,t}+\alpha_{5}{Pgdp}_{i,t}^{2}+\alpha_{6}{TrGl}_{i,t}+\alpha_{7}{FinGl}_{i,t}+\varepsilon_{i,t}\;;\;t=1,.\;.\;.,T;\;i=1,.\;.\;.,N;\;\varepsilon_{i,t}=\mu_{i}+u_{i,t}$$

As demonstrated in Equation (2), the BC-MM estimator enables the estimation of higher-order autoregressive models by incorporating p lags of the dependent variable. Unlike the IV and GMM estimators, the FE version of the BC-MM model serves as a just-identified method-of-moments estimator [58]. Therefore, the use of instruments or accompanying validations is not required [58]. To verify the absence of second-order serial correlation in the first-differenced residuals, we employ the test proposed by Arellano and Bond [59].

### 3.3 Definition of variables and data sources

This study utilizes annual panel data for 30 countries in Sub-Saharan Africa, covering the period from 1995 to 2019 (T = 25 and N = 30) (refer to Table A2 in the Appendix). There are two relevant aspects of DSD discussed in the literature. The first aspect involves disaggregating the overall economic output based on sectorial contributions [60]. In this study, the output composition of primary industry (Agriculture, forestry, and fishing, value added as a percentage of GDP) (PVA), secondary industry (Construction, Mining, Manufacturing, and Energy & Water Utilities Sectors, value added as a percentage of GDP) (SVA), and tertiary industry (Services, value added as a percentage of GDP) (TVA) are considered crucial for understanding DSD in the SSA sub-region. Based on data availability, SVA is further disaggregated into manufacturing (MVA) and the sub-sectors of mining, construction, and utilities (MCU).

The second approach to defining DSD involves tracking the transformation of the industrial structure towards a service-based economy (STR). This is computed using the following formula [61,62]:

(3) $$STR_{t}=\sum_{t=1}^{3}Y_{t}\times i=PVA+MCU+2*MVA+3*TVA$$

The maximum value that STR can reach is three, indicating that the tertiary industry accounts for the entire GDP. Conversely, the minimum value is one, indicating that agriculture, forestry, and fishing, mining, construction, and utilities sectors comprise the entire GDP share. A higher STR value signifies a transition in the composition of the industrial structure towards a service-oriented economy, reflecting progress in industrial structure transformation.

Two explanatory variables are the focus of this study: economic dependence on natural resources (NR) and financial development (FD). Consistent with the relevant literature, NR is defined as the sum of economic rents derived from oil, coal, natural gas, forest and minerals, expressed as a percentage of GDP. A higher NR value indicates a greater reliance of the economy on rents from natural resources. FD is measured using a comprehensive index of financial development compiled by Svirydzenka [15]. This index tracks the developmental trends of the financial system, encompassing depth, access, and efficiency indicators. The data series and their notations include: FI - development of the financial institutions segment (including banking and non-banking institutions); FM - development of the financial markets segment; FD - an overall index reflecting the development trends in the financial system based on FI and FM indicators. These indices are normalized on a scale from 0 (representing the lowest level of development) to 1 (representing the highest level of development). To account for the multidimensional nature of financial development, the depth (FID), access (FIA), and efficiency (FIE) indicators of the FI category within the FD dataset are considered. The motivation behind our focus on FI development stems from the relatively low level of FM development in SSA [15], which results in a lack of data availability for the efficiency and access sub-indices of FM in most countries within the panel constructed for this study.

In accordance with existing literature, per capita income is represented by real per capita GDP (Pgdp). The Trade Globalization (TrGl) index combines both de jure and de facto characteristics that influence cross-border trade flows [63]. Similarly, the Financial Globalization (FinGl) index combines both de jure and de facto characteristics that influence cross-border investment and capital flows [63]. Table 1 provides a summary description of the variables and their data sources. Additionally, Table 2 presents the summary statistics for these variables. The average STR value is 1.918, with minimum and maximum ratios of approximately 1.226 and 2.358, respectively. These estimates suggest that, on average, structural transformation has been slow in sub-Saharan Africa, with the primary and mineral extraction sectors continuing to play a dominant role in driving economic activity within the region.

**Table 1:** Definition of variables and data sources.

Variables	Definition	Data Sources
PVA	Agriculture, forestry, and fishing, value added (% of GDP)	The World Development Indicators (WDI), World Bank
SVA	Industry (including construction), value added (% of GDP)	WDI, World Bank
TVA	Services, value added (% of GDP)	WDI, World Bank
MVA	Manufacturing, value added (% of GDP)	WDI, World Bank
MCU	Mining, Construction and electricity, water, and gas utilities, value added (% of GDP)	WDI, World Bank
STR	Industrial structure upgrading	Computed based on Eq. (3) using data from WDI Database, World Bank
NR	Total natural resources (sum of Oil, Coal, natural gas, forest and mineral) rents (% of GDP)	WDI Database, World Bank
FD	Index of Financial development	IMF Financial Development Database
FI	Index of Financial Institutions Development	IMF Financial Development Database
FID	Financial Institutions Depth Index	IMF Financial Development Database
FIE	Financial Institutions Efficiency Index	IMF Financial Development Database
FIA	Financial Institutions Access Index	IMF Financial Development Database
FM	Index of Financial Markets Development	IMF Financial Development Database
Pgdp	GDP per capita (constant 2015 US$)	WDI Database, World Bank
TrGI	Index of Trade Globalization	KOF Swiss Economic Institute
FinGI	Index of Financia Globalization	KOF Swiss Economic Institute

**Table 2:** Basic descriptive statistics.

Variables	Mean	Median	Maximum	Minimum	Std. Dev.	Skewness	Kurtosis	Observations
PVA	22.042	21.883	60.611	1.798	14.216	0.427	2.478	750
SVA	26.219	23.700	72.717	4.556	12.749	1.258	4.394	750
MCU	15.621	11.276	69.495	1.561	13.232	1.838	6.029	750
MVA	10.598	9.597	35.215	0.233	5.892	1.704	7.412	750
TVA	44.319	44.648	70.337	12.435	9.481	−0.011	3.278	750
STR	1.918	1.937	2.358	1.226	0.212	−0.223	2.863	750
NR	12.237	9.003	58.688	0.066	11.143	1.630	5.583	750
FD	0.144	0.106	0.643	0.003	0.112	2.260	8.234	750
FI	0.219	0.175	0.731	0.004	0.140	1.958	6.383	750
FID	0.122	0.058	0.877	0.001	0.186	2.866	10.356	750
FIE	0.497	0.502	0.859	0.005	0.136	−0.513	3.390	750
FIA	0.089	0.042	0.899	0.003	0.141	3.279	14.680	750
FM	0.064	0.013	0.535	0.000	0.101	2.248	8.061	750
Pgdp	2024.644	932.689	16989.960	219.193	2690.642	2.688	11.161	750
TrGl	40.618	39.404	81.594	13.200	12.268	0.515	3.396	750
FinGl	45.758	47.189	75.998	21.237	11.432	0.074	2.443	750

## 4 Presentation and discussion of results

### 4.1 Preliminary evidence from exploratory analysis

**Fig. 1:**
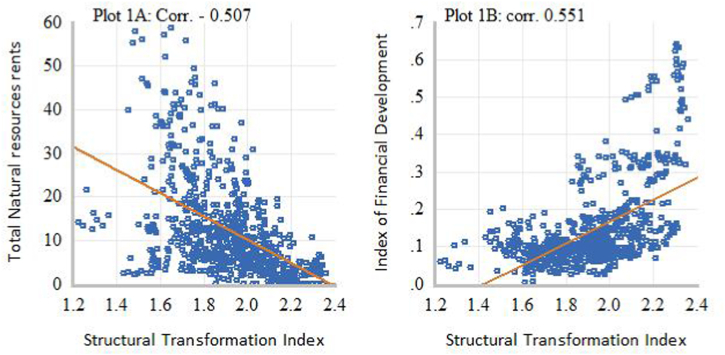
Scatter plots of linear association between STR and NR (Plot A) and FD (Plot B).

**Fig. 2:**
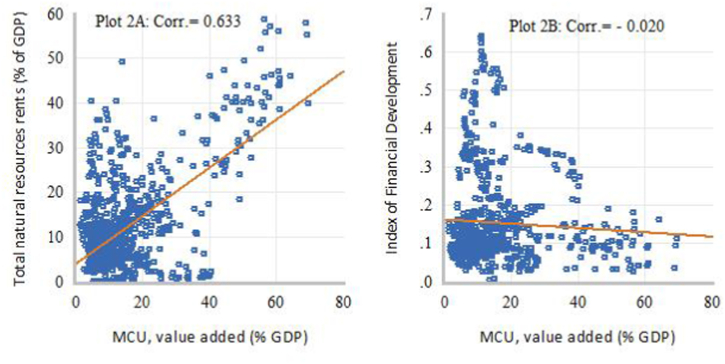
Scatter plots of linear association between MCU and NR (Plot 2A) and FD (Plot 2B).

**Fig. 3:**
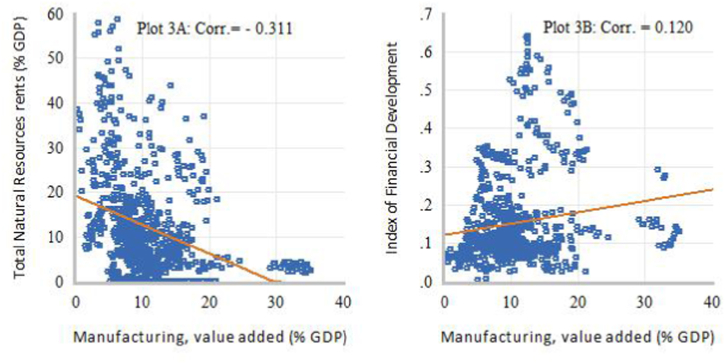
Scatter plots of linear association between MVA and NR (Plot 3A) and FD (Plot 3B).

**Fig. 4:**
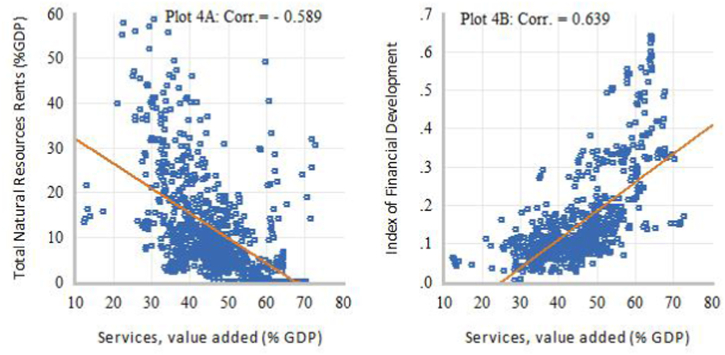
Scatter plots of linear association between TVA and NR (Plot 4A) and FD (Plot 4B).

Fig. 1, Fig. 2, Fig. 3, Fig. 4 present some exploratory analysis based on scatter plots with a linear fitted line, illustrating the relationship between industrial structure indicators (DSD), intensity of natural resource dependence (NR), and financial development (FD) in SSA during the study period. In Fig. 1A, it is evident that STR and NR display a negative co-movement, with a correlation of −0.507. Conversely, Fig. 1B reveals that STR and FD exhibit a positive co-movement, showing a correlation of 0.551. When the secondary industry is disaggregated into MCU and manufacturing, marked differences in the degree of linear association can be observed. For example, Fig. 2A demonstrates a positive correlation of 0.633 between MCU and NR, while Fig. 2B shows a weak negative co-movement, with a correlation of −0.020, between MCU and FD. This means that the relationship between natural resource rents and the added output value of the secondary industry is primarily driven by activities in the MCU sectors. On the other hand, Fig. 3A displays a negative correlation of −0.311 between MVA and NR, whereas Fig. 3B illustrates a positive co-movement with a correlation of 0.120 between MVA and FD. Lastly, Fig. 4A depicts a negative co-movement with a correlation of −0.589 between TVA and NR, while Fig. 4B exhibits a positive co-movement, revealing a correlation of 0.639 between TVA and FD. These findings from the exploratory analyses reinforce the view that economic reliance on natural resources affects the composition and transformation of industrial structure in SSA [2]. It also generates interest with a policy focus on identifying efficient resource allocation mechanisms that could deepen structural transformation towards technology-driven industrialization in SSA.

### 4.2 Results from dynamic panel model analysis

The results of the initial analysis are presented in Table 3, grouped into panels. The empirical steps rely on the more robust fixed effects (FE) version of the BC-MM estimator, which generates efficiently-identified parameter estimates and does not require a preliminary consistent estimator [57]. The Arellano-Bond serial correlation test applied confirmed the absence of AR(2) in the first difference residuals in all specifications, indicating that the estimates are consistent and reliable for policy analysis. Each panel consists of two sets of estimates, with the estimates in the first column using the FD Composite Index to define the impact of financial development. The estimates in the second column decompose the FD index into FI and FM. Panel A estimates explain the output value of the primary sector, which includes agriculture, forestry, and fishing industries (i.e., DSD = PVA). The results indicate that only TrGl offers a statistically significant explanation for PVA in the selected SSA countries. The coefficient estimates show that PVA decreases by 0.043% for a 1% increase in the degree of trade globalization. This finding deviates from the positive effect reported by Alagidede et al. [48] in a sample of 28 SSA countries for the period 1985–2015. The use of more recent data for this study suggests the evolving transformation-inducing role of economic globalization in SSA.

**Table 3:** Impact of NRR and FD on Primary, Secondary and Tertiary sectors.

Variables	(1)	(2)	(3)	(4)	(5)	(6)	(7)	(8)
	Panel A: DSD = PVA		Panel B: DSD = MCU		Panel C: DSD = MVA		Panel D: DSD = TVA	
L.DSD	0.893***	0.897***	0.843***	0.840***	0.804***	0.804***	0.716***	0.715***
	(0.141)	(0.151)	(0.038)	(0.038)	(0.070)	(0.069)	(0.040)	(0.042)
NR	−0.015	−0.015	0.072**	0.072**	−0.084*	−0.084*	−0.073***	−0.073***
	(0.025)	(0.026)	(0.031)	(0.031)	(0.050)	(0.050)	(0.018)	(0.017)
FD	−0.003		−0.009		0.103***		0.022*	
	(0.056)		(0.033)		(0.036)		(0.012)	
FI		0.005		0.003		0.099***		0.026**
		(0.057)		(0.028)		(0.037)		(0.011)
FM		0.000		−0.005*		0.004		0.001
		(0.002)		(0.003)		(0.003)		(0.001)
Pgdp	0.059	0.062	0.647**	0.664**	−0.019	0.039	−0.527***	−0.512***
	(0.170)	(0.173)	(0.280)	(0.279)	(0.252)	(0.257)	(0.133)	(0.133)
Pgdp^2^	−0.006	−0.006	−0.034**	−0.035**	−0.013	−0.018	0.035***	0.034***
	(0.011)	(0.011)	(0.017)	(0.017)	(0.013)	(0.014)	(0.010)	(0.010)
TrGl	−0.042**	−0.043*	0.003	0.014	−0.120**	−0.117**	0.040	0.041
	(0.019)	(0.023)	(0.050)	(0.053)	(0.047)	(0.047)	(0.028)	(0.029)
FinGl	0.066	0.066	0.069	0.063	0.017	0.025	−0.069*	−0.067*
	(0.047)	(0.046)	(0.070)	(0.069)	(0.064)	(0.063)	(0.037)	(0.036)
Constant	0.101	0.091	−2.826***	−2.902***	1.974	1.727	3.256***	3.211***
	(0.576)	(0.566)	(1.051)	(1.053)	(1.279)	(1.259)	(0.507)	(0.500)
Turning point Pgdp	–	–	9.515	9.486	–	–	7.529	7.529
Turning point Pgdp in US$	–	–	13557.65	13170.23	–	–	1860.45	1862.01
Arellano–Bond Test for AR(2): Prob > z	0.415	0.462	0.147	0.160	0.263	0.269	0.479	0.454
Time effects	Yes	Yes	Yes	Yes	Yes	Yes	Yes	Yes
Observations	720	720	690	690	690	690	690	690
Number of instruments	–	–	–	–	–	–	–	–
Number of ID	30	30	30	30	30	30	30	30

The results in Panel B explain the output value of the mining, construction, and utilities (electricity, water, and gas) industries (DSD = MCU). The coefficient of NR indicates that resource dependence has a significant positive effect, increasing the industrial output value share of the MCU sectors by 0.072% for a 1% increase in derived rents (see column 6). This clearly indicates a resource-based path to industrialization and supports the growing empirical illustration of resource curse symptoms in SSA [2,42]. FD exerts a significant negative impact on MCU through the FM segment, leading to a 0.005% decrease in MCU output value for every 1% increase in financial markets development. This finding reinforces the potential of financial markets to mitigate the dominance of extractive and non-tradable industries and alleviate the associated symptoms of the resource curse. Pgdp positively influences MCU, while the squared term, Pgdp^2^, exhibits a negative impact, with both estimates being statistically significant. These findings suggest a valid inverted U-shaped relationship between Pgdp and the value added of the MCU sectors. Specifically, MCU tends to increase at lower levels of Pgdp and decrease at higher levels. From a policy perspective, it is predicted that the contribution of MCU to aggregate output will decline once Pgdp reaches the turning point level of US$13,170.23 (refer to column 4).

The results in Panel C elucidate the output value of the manufacturing component of the secondary sector (DSD = MVA). The parameter estimates reveal a significant negative impact of NR on MVA, indicating that a 1% increase in NR leads to a decrease in MVA by 0.084%. This finding confirms the crowding-out effect of natural resource dependence on manufacturing output and substantiates the presence of Dutch disease symptoms in SSA, as previously reported by Asiamah et al. [2]. These results are also consistent with the findings of Haraguchi et al. [44] and Alssadek and Benhin [35] in other developing economies with abundant natural resources. The impact of financial development on MVA is positive and depends significantly on the development trends in the financial institutions sector. The coefficient of FI indicates that a 1% increase in financial institutions development results in a 0.099% increase in MVA. This finding contrasts with the earlier study by Itaman and Awopegba [16], which reported a negative effect on manufacturing output value in Nigeria. Comparatively, FD primarily influences the output value of the secondary sector through MVA, thereby generating interest in its role in reallocating resources from primary, extractive, and non-tradable industries to the more growth-enhancing manufacturing industry [38]. These results align with the findings of Choi [39] and Hasanov et al. [36] regarding the role of FD in mitigating the structural consequences of the resource curse. The impact of Pgdp and its square term on MVA is found to be statistically insignificant, which aligns with the earlier findings by Botta et al. [5] for African EDEs. However, these results differ from the findings of Dabla-Norris et al. [11] from a sample of developing economies. Economic globalization has a significant impact on MVA through the TrGl pathway, with a 1% increase in trade globalization leading to a 0.117% decrease in MVA. While Alagidede et al. [48] also found a significant role for TrGl, their study suggests a positive impact in SSA, which contrasts with our findings.

The results in Panel D explain the output value of the tertiary (services) sector (i.e., DSD = TVA). The findings indicate a significant negative effect of NR on TVA, with a 1% increase in NR leading to a decrease in TVA by 0.073% (see column 8). This confirms the crowding-out effect of natural resources on productive sectors in SSA, as previously reported by Asiamah et al. [2] and Nkemgha et al. [45]. On the other hand, FD has a positive impact on TVA, primarily through the path of financial institutions development. The estimates show that a 1% increase in FI results in a 0.026% increase in TVA. Pgdp has a negative impact on TVA, while the square term has a positive impact, both statistically significant. These findings indicate a valid U-shaped relationship between Pgdp and TVA, with a calculated turning point level of Pgdp at US$1862.01. This suggests that a shift towards the services sector does not necessarily imply premature termination of the industrialization process, as suggested by some studies in the literature [24]. As highlighted by Sen [18], the emergence of digital technologies has transformed the services sector, enabling it to provide similar features of manufacturing-led industrialization that facilitated scale, innovation, and spill-over effects in the now-industrialized countries. The impact of FinGl on TVA is negative and significant, highlighting another pathway through which economic globalization weakens structural diversification SSA.

The results presented in Table 4 explain the process of structural transformation in SSA by utilizing a metric that tracks the shifts in the composition of aggregate output towards tertiary production (DSD = STR). To ensure the robustness of our analysis, we employ alternative estimators (System GMM and IV-DFreg) in addition to the FE-BC-MM approach. The derived parameter estimates are complemented by essential post-estimation validation tests, including the Arellano-Bond AR(2) serial correlation test, as well as the Sargan-test, Hansen-test, and Diff-in-Hansen test to assess the validity of the instruments. The results indicate the rejection of the presence of AR(2) in the first difference residuals, affirming the consistency and reliability of our estimates. Furthermore, the tests confirm the validity of the instruments across all specifications. To prevent over-specification, we follow the suggestion of Roodman [54] and ensure that the number of instruments used is less than the number of cross-sectional groups.

**Table 4:** Impact of NRR and FD on Structural transformation (DSD = STR).

Variables	(1)	(2)	(3)	(4)	(5)	(6)	(7)	(8)	(9)	(10)
	BC-MM Estimation with fixed effects				Robustness check using alternative estimators					
	AR(1) dynamics	AR(1) dynamics	AR(2) dynamics	AR(4) dynamics	one-step system GMM	one-step system GMM	two-step system GMM	two-step system GMM	IV-DFreg	IV-DFreg
L.STR	0.699***	0.697***	0.734***	0.683***	0.674***	0.672***	0.668***	0.690***	0.291***	0.298***
	(0.032)	(0.033)	(0.031)	(0.036)	(0.064)	(0.059)	(0.063)	(0.045)	(0.039)	(0.029)
NR	−0.034***	−0.035***	−0.035***	−0.036***	−0.037**	−0.039***	−0.028*	−0.028**	−0.037***	−0.046***
	(0.007)	(0.007)	(0.008)	(0.007)	(0.014)	(0.014)	(0.014)	(0.011)	(0.007)	(0.004)
FD	0.008				0.063***		0.070***		0.021***	
	(0.009)				(0.021)		(0.023)		(0.003)	
FI		0.013**	0.014**	0.017**		0.061***		0.069***		0.020***
		(0.006)	(0.006)	(0.007)		(0.017)		(0.017)		(0.002)
FM		0.001	0.001	0.002***		−0.001		−0.001		0.002***
		(0.001)	(0.000)	(0.000)		(0.001)		(0.002)		(0.001)
Pgdp	−0.225***	−0.217***	−0.224***	−0.234***	0.145	0.144	−0.019	0.001	−1.051***	−0.725***
	(0.060)	(0.059)	(0.057)	(0.067)	(0.250)	(0.262)	(0.211)	(0.179)	(0.323)	(0.064)
Pgdp^2^	0.015***	0.015***	0.015***	0.015***	−0.011	−0.010	0.001	−0.000	0.059***	0.039***
	(0.004)	(0.004)	(0.004)	(0.005)	(0.017)	(0.018)	(0.015)	(0.012)	(0.022)	(0.005)
TrGl	0.007	0.006	0.004	−0.012	−0.076	−0.069	−0.086*	−0.073**	−0.014	0.002
	(0.013)	(0.013)	(0.013)	(0.011)	(0.045)	(0.045)	(0.047)	(0.034)	(0.016)	(0.012)
FinGl	−0.032*	−0.031*	−0.028*	−0.026	−0.084**	−0.071*	−0.074*	−0.065*	−0.025	−0.023**
	(0.017)	(0.016)	(0.016)	(0.016)	(0.041)	(0.037)	(0.040)	(0.035)	(0.016)	(0.012)
Constant	1.172***	1.152***	1.198***	1.358***	0.526	0.408	1.114	0.892	5.167***	3.809***
	(0.247)	(0.244)	(0.218)	(0.285)	(0.943)	(0.922)	(0.827)	(0.611)	(1.328)	(0.303)
Turning point Pgdp	7.500	7.233	7.467	7.800					8.907	9.295
Turning point Pgdp in US$	1808.04	1384.83	1748.77	2440.60					7381.85	10882.07
Arellano–Bond Test for AR(2): Prob > z	0.366	0.321	0.439	0.951	0.227	0.221	0.226	0.231	–	–
Sargan test: Prob > chi2	–	–	–	–	0.236	0.427	0.236	0.427	–	–
Hansen test: Prob > chi2	–	–	–	–	0.549	0.747	0.549	0.747	0.301	0.485
Difference-in-Hansen: Prob > chi2	–	–	–	–	0.728	0.804	0.728	0.804	–	–
Time effects	Yes	Yes	Yes	Yes	–	–	–	–	–	–
Observations	720	720	690	630	630	630	630	630	660	660
Number of instruments	–	–	–	–	16	18	16	18	24	28
Number of ID	30	30	30	30	30	30	30	30	30	30

All the estimators yield consistent results in terms of the signs and statistical properties of the coefficient estimates for NR and FD. Among the estimators used, we rely on the more robust estimates provided by the FE-BC-MM estimator (see columns 1–4). The coefficient of NR is consistently negative and statistically significant across the estimations, indicating that a 1% increase in NR leads to a decrease in STR by 0.034%–0.036%. This suggests that natural resource dependence hampers the transformation of the industrial structure towards the more growth-enhancing manufacturing and tertiary sectors. Similar empirical evidence has been documented by Amiri et al. [43] for a selection of 28 natural resource-dependent countries and by Wu et al. [9] for China. On the other hand, FD has a positive impact on STR, particularly through the path of financial institutions development. The estimates reveal that a 1% increase in FI leads to a 0.013%–0.017% shift in the industrial structure, favouring the growth of the manufacturing and tertiary sectors. This finding implies that financial development facilitates the process of industrial transformation. Examining the construction of the STR indicator allows us to identify the underlying pathway through which FD exerts its transformation-inducing effects. As observed from the results in Table 3, FD stimulates the expansion of aggregate output by promoting value additions in manufacturing and services. Consequently, FD mitigates the crowding-out effect of natural resources on the manufacturing and tertiary sectors. These results align with the findings of Wang and Wang [47] and Jiang et al. [46] in China, as well as the Taiwanese firm-level evidence presented by Choi [39] and the findings of Hasanov et al. [36] in Azerbaijan and Kazakhstan.

Regarding Pgdp, it has a negative impact on STR, while the square term exhibits a positive impact, with both being statistically significant. These estimates indicate the presence of a valid U-shaped curve between Pgdp and STR, implying that per capita income is positively correlated with structural transformation, but only after a certain threshold level of income is reached. The estimated turning point level of income is higher than the most recent (2021) World Bank estimate for the region, which stands at US$1599.20 [1]. This suggests that the region has yet to reach the turning point in the curve. The coefficient of FinGl is negative and significant, indicating that financial globalization weakens the process of industrial structure transformation in SSA. Consequently, addressing the structural challenges caused by the reliance on abundant natural resources may require focusing on domestic policy channels such as the development of the financial sector.

In the empirical estimation of the results presented in Table 3, Table 4, we utilized aggregated indices to approximate financial development, considering the development trends in financial institutions and markets. However, for a more comprehensive policy analysis, we further disaggregated the effect of financial development, considering its complex multidimensional nature, including depth, access, and efficiency. Table 5 provides the results from the disaggregated analysis of the role of financial institutions (FI). The analysis reveals that Financial Institutions Depth (FID) has a significant positive impact on Manufacturing Value Added (MVA), while its impact on Mining, Construction, and Utilities (MCU) is statistically insignificant. The estimated elasticity coefficient suggests that a 1% increase in FID leads to a 0.068% increase in MVA. Furthermore, Financial Institutions Efficiency (FIE) has a significant positive impact on both MVA and TVA, thereby facilitating a deeper shift in the industrial structure away from MCU. According to the coefficient estimates, a 1% increase in FIE results in a 0.069% increase in MVA and a 0.017% increase in TVA. Consequently, the industrial structure experiences a 0.010% shift away from primary and extractive-based industries towards the more growth-enhancing manufacturing and services sectors. These findings emphasize the importance of enhancing both the depth and efficiency of the financial systems in promoting structural diversification in SSA. Even if financial institutions have broad reach and depth, their contribution to structural diversification would be limited if they operate inefficiently. This point is supported by earlier findings reported by Choi [39].

**Table 5:** Disaggregated FD analysis (using FE-BC-MM estimator).

Variables	(1)	(2)	(3)	(4)	(5)	(6)	(7)	(8)	(9)	(10)	(11)	(12)
	Financial Institutions Depth				Financial Institutions Efficiency				Financial Institutions Access			
	DSD = MCU	DSD = MVA	DSD = TVA	DSD = STR	DSD = MCU	DSD = MVA	DSD = TVA	DSD = STR	DSD = MCU	DSD = MVA	DSD = TVA	DSD = STR
L.DSD	0.842***	0.795***	0.716***	0.736***	0.841***	0.806***	0.735***	0.734***	0.839***	0.807***	0.718***	0.737***
	(0.038)	(0.075)	(0.041)	(0.031)	(0.038)	(0.070)	(0.036)	(0.031)	(0.039)	(0.074)	(0.043)	(0.031)
NR	0.074**	−0.076	−0.072***	−0.035***	0.072**	−0.085*	−0.071***	−0.035***	0.071**	−0.082	−0.073***	−0.035***
	(0.030)	(0.048)	(0.018)	(0.008)	(0.030)	(0.051)	(0.016)	(0.008)	(0.030)	(0.050)	(0.018)	(0.008)
FID	0.017	0.068**	0.012	0.003								
	(0.034)	(0.030)	(0.016)	(0.006)								
FIE					0.010	0.069***	0.017**	0.010*				
					(0.020)	(0.026)	(0.008)	(0.006)				
FIA									−0.014	0.021	0.000	−0.001
									(0.016)	(0.019)	(0.015)	(0.008)
FM	−0.004	0.005*	0.001	0.001	−0.005*	0.004	0.002	0.001	−0.005*	0.002	0.001	0.000
	(0.004)	(0.003)	(0.001)	(0.001)	(0.003)	(0.003)	(0.001)	(0.001)	(0.003)	(0.003)	(0.001)	(0.000)
Pgdp	0.647**	−0.080	−0.544***	−0.238***	0.669**	−0.017	−0.465***	−0.232***	0.705***	−0.115	−0.535***	−0.234***
	(0.297)	(0.296)	(0.133)	(0.058)	(0.278)	(0.259)	(0.130)	(0.059)	(0.264)	(0.285)	(0.142)	(0.059)
Pgdp^2^	−0.033*	−0.010	0.036***	0.016***	−0.035**	−0.014	0.032***	0.015***	−0.038**	−0.008	0.035***	0.015***
	(0.019)	(0.018)	(0.010)	(0.004)	(0.017)	(0.014)	(0.010)	(0.004)	(0.016)	(0.016)	(0.011)	(0.005)
TrGl	0.006	−0.146***	0.037	0.003	0.013	−0.116**	0.043	0.004	0.014	−0.112**	0.042	0.004
	(0.050)	(0.052)	(0.031)	(0.014)	(0.053)	(0.048)	(0.027)	(0.014)	(0.054)	(0.053)	(0.030)	(0.014)
FinGl	0.057	0.004	−0.071*	−0.029*	0.063	0.028	−0.069**	−0.028*	0.065	0.019	−0.067*	−0.028*
	(0.069)	(0.064)	(0.038)	(0.017)	(0.069)	(0.066)	(0.035)	(0.016)	(0.067)	(0.062)	(0.038)	(0.016)
Constant	−2.771**	2.336	3.343***	1.242***	−2.923***	1.774	2.882***	1.205***	−3.108***	2.194	3.249***	1.208***
	(1.147)	(1.471)	(0.514)	(0.224)	(1.043)	(1.277)	(0.509)	(0.226)	(0.944)	(1.388)	(0.536)	(0.217)
Turning point Pgdp	9.803	–	7.556	7.438	9.557	–	7.266	7.733	9.276	–	7.643	7.800
Turning point Pgdp in US$	18088.48	–	1911.33	1698.50	14145.37	–	1430.28	2283.20	10682.00	–	2085.69	2440.60
Time effects	Yes	Yes	Yes	Yes	Yes	Yes	Yes	Yes	Yes	Yes	Yes	Yes
Arellano–Bond Test for AR(2): Prob > z	0.160	0.301	0.625	0.570	0.157	0.267	0.478	0.440	0.162	0.284	0.623	0.573
Observations	690	690	690	690	690	690	720	690	690	690	690	690
Number of ID	30	30	30	30	30	30	30	30	30	30	30	30

## 5 Conclusion and policy direction

As the economy evolves, it shifts from agriculture and mineral extraction to more manufacturing and services. In the Sub-Saharan African (SSA) economies, a key policy focus is on the role of natural resources. With the abundance of natural resources in the region, ranging from oil, natural gas, minerals, and forests, it was anticipated that SSA countries would rapidly evolve into industrialized economies. Today, the structural features of the economies still remain unchanged, with heavy reliance on agriculture and mineral extraction as significant contributors to GDP. Against this backdrop, this study provides empirical evidence to guide policy considerations targeting structural diversification in the economies. Using a dynamic panel-data model and the newly developed bias-corrected method-of-moments estimator, this study examines the impact of natural resource dependence on industrial structure dynamics, with extended equations that incorporate the role of financial development. The composition of the empirical analysis relied on a panel of 30 countries selected based on data availability for the period 1995–2019.

The analysis yields several empirical findings. First, economic dependence on natural resources positively affects the combined output value of mining, construction, and utilities supply industries. However, it negatively and significantly impacts the output value of the manufacturing and services industries. These findings indicate that natural resource dependence hinders the shift towards a manufacturing and services-oriented industrial structure in SSA. Second, financial development, particularly through the institutions segment, negatively impacts the combined output value of mining, construction, and utilities supply industries. Conversely, it positively and significantly affects the output value of the manufacturing and services industries. As a result, it contributes to a net positive effect on the evolution of industrial structure, fostering a shift away from extractive-based industrialization. Additionally, the study highlights the multidimensional nature of financial development, underscoring the importance of improving the depth and efficiency of the system in fostering structural diversification in SSA. Third, a relationship characterized by an inverted U-shaped curve exists between per capita GDP and the combined output value of mining, construction, and utilities industries. Conversely, the shift away from agriculture and extractive industries towards manufacturing and services follows a U-shaped curve with respect to per capita GDP. These findings reveal that economic growth positively influences industrial structure transformation only after reaching a certain threshold level of income per capita. Finally, the results demonstrate that economic globalization, specifically through trade and financial channels, weakens structural diversification in SSA.

The above findings hold significant policy implications. One, in line with the 2030 agenda for sustainable development, industrialization in SSA must be accompanied by structural transformation. This is evident from the observed U-shaped curve between per capita GDP and the structural shifts in the composition of industrial output. Achieving inclusive and sustainable development in SSA necessitates the spill-over effects, productive linkages, and growth in innovation and technology diffusion that arise from the process of industrial structure transformation. Two, economic dependence on natural resources hinders the progress of industrial structure evolution towards technologically-driven and growth-enhancing manufacturing and tertiary industries. Consequently, deeper structural transformation in SSA requires addressing institutional, political, and economic conditions that foster rent-seeking behaviour and undermine the drive for diversification. Three, financial development can support economic efficiency and diversification strategies by influencing the allocation of resources within the economy. Thus, the interaction between the financial sector and the real economy plays a crucial role in facilitating the transformation of industrial structure away from low-productivity (agriculture and extractive) industries towards more efficient and high-productivity manufacturing and tertiary industries. However, for the financial systems to have a meaningful impact, they must be efficient and improve on all aspects of operational effectiveness, while also avoiding wasteful practices. Therefore, policy considerations should focus on enhancing the depth and efficiency of intermediating savings into investments. Policymakers should target reducing the lending-deposit spread and overhead costs of operation, which are key indicators of efficiency loss in financial intermediation.

Future research endeavours can aim to identify additional policies that can assist developing economies in navigating the challenges associated with economic dependence on natural resources and promoting sustainable development. It is crucial to explore emerging areas of interest, such as digital technologies, renewable energy, and climate considerations, to understand their roles in shaping structural transformation.

## Author contribution statement

Chinazaekpere Nwani: conceived and designed the study; analyzed and interpreted the data; wrote the paper.

Benedette Nneka Okezie: wrote the paper; contributed materials, & analysis tools.

Anthony Chukwuma Nwali: wrote the paper; contributed materials, & analysis tools or data.

Johnson Nwokeiwu: wrote the paper; contributed materials, & analysis tools or data.

Gloria Ifeoma Duruzor: wrote the paper; contributed materials, & analysis tools or data.

Ogbonna Nweze Eze: wrote the paper; contributed materials, & analysis tools or data.

## Data availability statement

Data associated with this study has been deposited at The World Development Indicators (WDI) available online: https://databank.worldbank.org/source/world-development-indicators/preview/on; IMF Financial Development Database is available online: https://data.imf.org/?sk=F8032E80-B36C-43B1-AC26-493C5B1CD33B; KOF Globalization Index available online: https://kof.ethz.ch/en/forecasts-and-indicators/indicators/kof-globalisation-index.html.

## Declaration of competing interest

The authors declare that they have no known competing financial interests or personal relationships that could have appeared to influence the work reported in this paper.

## Figures and Tables

**Table A2:** Hausman specification test

DSD Specifications	chi2	Prob > chi2	$H_{0}$: Difference in coefficients not systematic
Agriculture, forestry, and fishing (PVA)	16.14**	0.0234	Reject $H_{0}$; FE model is more efficient
Industry, including mining and construction (SVA)	39.77***	0.000	Reject $H_{0}$; FE model is more efficient
Manufacturing (MVA)	26.58***	0.000	Reject $H_{0}$; FE model is more efficient
Services (TVA)	66.28***	0.000	Reject $H_{0}$; FE model is more efficient
Industrial structure upgrading (STR)	46.12***	0.000	Reject $H_{0}$; FE model is more efficient

Table A2. Sample Countries			
Angola	Cote d'Ivoire	Mozambique	Togo
Benin	Eswatini	Namibia	Uganda
Botswana	Ethiopia	Niger	Zambia
Burkina Faso	Gabon	Nigeria	
Burundi	Ghana	Rwanda	
Cameroon	Guinea	Seychelles	
Chad	Kenya	Sierra Leone	
Congo Dem. Rep	Lesotho	South Africa	
Congo Rep.	Malawi	Tanzania	
